# Interleukin-1α Mediates Ozone-Induced Myeloid Differentiation Factor-88-Dependent Epithelial Tissue Injury and Inflammation

**DOI:** 10.3389/fimmu.2018.00916

**Published:** 2018-05-07

**Authors:** Chloé Michaudel, Isabelle Maillet, Louis Fauconnier, Valérie Quesniaux, Kian Fan Chung, Coen Wiegman, Daniel Peter, Bernhard Ryffel

**Affiliations:** ^1^Laboratory of Experimental and Molecular Immunology and Neurogenetics (INEM), UMR 7355 CNRS-University of Orleans, Orleans, France; ^2^ArtImmune SAS, Orleans, France; ^3^Airways Disease, National Heart and Lung Institute, Imperial College London, London, United Kingdom; ^4^NIHR Respiratory Biomedical Research Unit, Royal Brompton & Harefield NHS Foundation Trust, London, United Kingdom; ^5^Immunology and Respiratory Diseases Research, Boehringer Ingelheim Pharma GmbH & Co. KG, Biberach an der Riss, Germany

**Keywords:** ozone, interleukin-1α, interleukin-18, interleukin-1β, myeloid differentiation factor-88, epithelial cell, inflammation

## Abstract

Air pollution associated with ozone exposure represents a major inducer of respiratory disease in man. In mice, a single ozone exposure causes lung injury with disruption of the respiratory barrier and inflammation. We investigated the role of interleukin-1 (IL-1)-associated cytokines upon a single ozone exposure (1 ppm for 1 h) using IL-1α-, IL-1β-, and IL-18-deficient mice or an anti-IL-1α neutralizing antibody underlying the rapid epithelial cell death. Here, we demonstrate the release of the alarmin IL-1α after ozone exposure and that the acute respiratory barrier injury and inflammation and airway hyperreactivity are IL-1α-dependent. IL-1α signaling *via* IL-1R1 depends on the adaptor protein myeloid differentiation factor-88 (MyD88). Importantly, epithelial cell signaling is critical, since deletion of MyD88 in lung type I alveolar epithelial cells reduced ozone-induced inflammation. In addition, intratracheal injection of recombinant rmIL-1α in MyD88^acid^ mice led to reduction of inflammation in comparison with wild type mice treated with rmIL-1α. Therefore, a major part of inflammation is mediated by IL-1α signaling in epithelial cells. In conclusion, the alarmin IL-1α released upon ozone-induced tissue damage and inflammation is mediated by MyD88 signaling in epithelial cells. Therefore, IL-1α may represent a therapeutic target to attenuate ozone-induced lung inflammation and hyperreactivity.

## Introduction

Ozone (O_3_) due to air pollution causes acute and chronic respiratory diseases and exacerbation of allergic asthma (1). Indeed, respiratory exposure to ozone induces airway hyperreactivity in humans and animals, cell death, inflammatory cell recruitment, and emphysema (2), and production of cytokines, including IL-6 (3), IL-33 (4), and chemokines, like CXCL1 [keratinocyte chemoattractant (KC)], MIP-2, and MCP-1 in the lung (5).

Toll-like receptors (TLR) signal *via* the adaptor protein myeloid differentiation factor-88 (MyD88) resulting in an inflammatory response in the lung (6–9). The TLR4 ligand hyaluronan released upon ozone exposure may activate TLR4 *via* MyD88 causing inflammation (9–11). However, MyD88 is also used by the interleukin (IL-1) receptor family, including IL-1R1, IL-18R, IL-33R, and IL-36R activating NF-κB inducing pro-inflammatory cytokines expression (12). A previous study showed that MyD88 is activated upon ozone exposure resulting in airway hyperreactivity and neutrophilic inflammation (7).

Epithelial cells represent the first cell type exposed to ozone and may be particularly crucial for inflammatory responses. Tight junction complexes control the permeability of respiratory epithelium. Ozone exposure causes disruption of tight junctions and epithelial desquamation (13). Epithelial cell death releases alarmins, such as IL-1α, IL-25, TSLP, or IL-33 (14) leading to chemokine production with the recruitment of neutrophils, ILC2, or lymphocytes (15). IL-1α, IL-1β, and IL-1R1 are involved in several models of lung inflammation such as in allergic asthma induced by ovalbumin (16, 17) or cigarette smoke/viral exacerbation models (18).

Upon ozone exposure the expression of IL-1α (19), IL-1β (20, 21), and IL-1R1-dependent signaling have been reported (22). A recent study demonstrated that ozone induced ocular conjunctivitis in mice and that addition of IL-1α further increased ozone-induced activation of cultured human conjunctival epithelial cells (23). Furthermore, administration of the IL-1 receptor antagonist attenuated ozone-induced inflammation in guinea pigs (24). IL-33, another IL-1 family member, released upon epithelial injury promotes inflammation during allergic asthma (16, 25) as well as in a cigarette smoke/viral challenge model (26). Increased lung inflammation upon ozone exposure in obese mice was IL-33 dependent (27), but we showed that IL-33 had a partial protective effect upon acute ozone exposure (28). IL-18, another IL-1 family member, is constitutively expressed in epithelial cells throughout the body (29) as well as in macrophages and dendritic cells. IL-18 has a pro-inflammatory role, but its role in ozone-induced inflammation is unknown (30–32).

Here, we revisited the expression and roles of IL-1α, IL-1β, and IL-18 in the lung upon respiratory ozone exposure and the contribution of epithelial cells by using genetically modified mice and pharmacological tools. We report that alarmin IL-1α expression in lung upon ozone-induced epithelial cell injury and inflammation is dependent of type 1 alveolar epithelial cells *via* MyD88 signaling.

## Materials and Methods

### Mice and Reagents

Myeloid differentiation factor-88^−/−^(33), MyD88^flox/flox^ mice (kindly provided by Dr. Matthias Muller and Franco di Padova) were used to generate MyD88 x Acid/AQP5 cre mice, which lack MyD88 in alveolar epithelial type 1 cells (34) as described (6), IL-1α- and IL-1β-deficient mice were provided by Dr. Yoichiro Iwakura (35), IL-18^−/−^ from Jackson laboratory, and C57BL/6 littermate control (WT) mice were used for the study. Mice were housed and bred in pathogen-free animal facility at Transgenose Institute (TAAM-CNRS, UPS 44 under agreement D-45-234-6, 2014), Orleans, France. Mice were bred in a temperature controlled (23°C) facility with strict 12 h light/dark cycles and were given free access to food and water. Female mice (8–10-month-old) were used in this study. Animal experiments were performed with the approval of the French Institutional Ethical Committee under agreement CLE CCO 2015-1088.

To block IL-1α, we used an anti-mouse-IL-1α antibody (Clone ALF-161, eBioscience) and its isotype, armenian hamster IgG (clone eBio299Arm, eBioscience) by intra-peritoneal injection given at 200 μg/mouse, 12 h before ozone exposure.

Recombinant mouse IL-1α (rmIL-1α) (Biolegend 575004, 0.8 μg/mice) was instilled intratracheally (i.t.) 4 h after ozone exposure.

### Ozone-Induced Airway Inflammation

We used plexiglass chamber (EMB 104, EMMS^®^) to expose mice at 1 ppm for 1 h in all studies as described before (36). Ozone is generated from an ozonizer (Ozonizator Ozonizer S 500 mg, Sander^®^) and its level of 1.0 ppm was controlled by an ozone sensor (ATI 2-wire transmitter, Analytical Technology^®^). Mice were euthanized by progressive CO_2_ inhalation for 24 h after ozone exposure and bronchoalveolar lavage (BAL) was collected. Lungs were collected for further analyses after a cardiac perfusion with ISOTON II (acid-free balanced electrolyte solution Beckman Coulter, Krefeld, Germany).

### Bronchoalveolar lavage

After ozone exposure, BAL was performed by four lavages with 500 µL saline solution (NaCl 0.9%) *via* a cannula introduced into mice trachea. BAL fluids were centrifuged at 2,000 rpm for 10 min at 4°C, the supernatants were stored at −20°C for ELISA analysis. To prepare cytospin, we used pellets (Thermo Scientific, Waltham, MA, USA) from BAL, with Diff-Quik solution staining (Merz & Dade A.G., Dudingen Switzerland). Differential cell counts were performed with at least 400 cells per slide.

### Measurement of Mediators and Total Proteins

Bronchoalveolar lavage fluid was assessed for myeloperoxidase (MPO), KC, LCN2, MMP9, and TIMP1 concentration by ELISA (R&D systems, Abingdon, UK) according to the manufacturer’s instructions. Total protein levels in BALF were analyzed with the Bio-Rad DC Protein Assay. IL-1α, IL-1β, and IL-18 from lung homogenate (same part of lung) were measured by Luminex immunoassay (*Thermo Fisher Scientific*) using MagPix reader (Bio-Rad) according to the manufacturer’s instructions. Data were analyzed with Bio-Plex Manager software (Bio-Rad).

### Determination of Airway Hyperreactivity (AHR)

Airway Hyperreactivity was measured after inhalation of increasing concentrations of methacholine (5–40 mg/mL) and determination of airway resistance (RI) by invasive plethysmography using the FinePointe system (Buxco, DSI) after each concentration of methacholine, as previously described (25).

### Histology

The left lobe of lung was fixed in 4% buffered formaldehyde and paraffin embedded under standard conditions. Tissue sections (3 µm) were stained with hematoxylin and eosin. To assess histological changes, tissue sections were made (3 µm) with hematoxylin and eosin staining. Sections were blindly evaluated by to independant investigators with Nikon microscope (Nikon eclipse 80i, Country, magnification of 400x), following a semi-quantative severity score on five bronchi (0–3) for inflammatory cell infiltration and alveolar epithelial injury. Arrows indicate epithelial cell injury and asterisk, cell infiltration.

**Table:** 

Score	Inflammatory cell infiltration	Epithelial damage
0	No infiltration	No damage on epithelial cells
1	Little infiltration around vessels	Epithelial cells flattening
2	Little infiltration around vessels and bronchi	Complete epithelial cells flattening
3	High infiltration around vessels and bronchi	Desquamation of epithelial cells

### Flow Cytometry

Bronchoalveolar lavage cells were stained with CD45 (553081), EpCam (563478), and 7-AAD (559925) from BD Pharmingen during 25 min at 4°C in FACS buffer (PBS, 2% FCS, 2 mM EDTA). To gate epithelial cells, we used CD45^+^ and EpCam^+^ (Figure S1A in Supplementary Material).

### RNA Extraction, Reverse Transcription, and Quantitative Real-Time PCR

Total RNA was extracted from the same lung part using TRIzol reagent (Sigma), following by purification with RNeasy Mini Kit (Qiagen, Valencia, CA). Reverse transcription was performed on 0.9 µg of total RNA with GoScript™ Reverse Transcription System (Promega). The mRNA levels were examined by quantitative RT-PCR using the GoTaq^®^ qPCR Master Mix (Promega). The primer sequences used were: IL-1β (QT01048355), IL-1α (QT00113505), and IL-18 (QT00171129). Relative levels of mRNA expression were normalized to HPRT1 mRNA levels using a comparative method (2^−ΔCt^). Non-reverse-transcribed RNA samples and water were included as negative controls.

### Statistical Analysis

Data were analyzed using Prism version 7 (Graphpad Software, San Diego, CA, USA). The non-parametric Mann–Whitney test or the parametric one-way ANOVA tests with multiple Bonferroni’s comparison tests were performed. Values are expressed as mean ± SEM. Statistical significance was defined at a *p*-value <0.05: ****<0.0001, ***<0.001, **<0.01, *<0.05.

## Results

### IL-1α and IL-18 Are Induced After Ozone Exposure, but Not IL-1β

**Figure 1:**
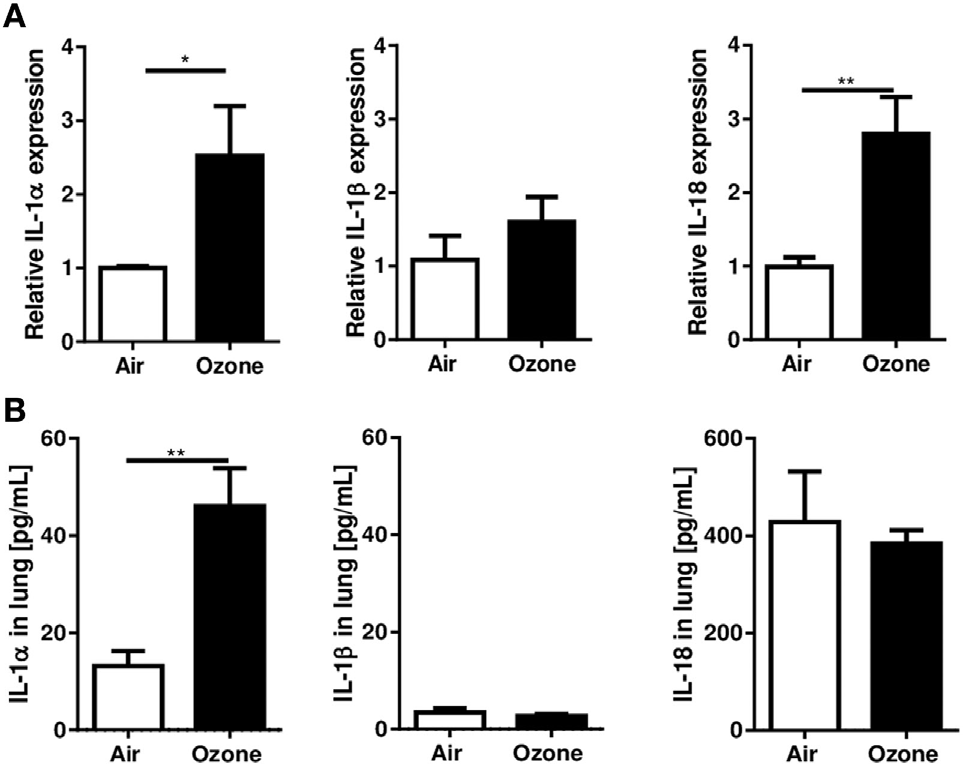
Interleukin (IL-1)α, IL-1β, and IL-18 expression in the lung 24 h after ozone exposure. Expression of IL-1α, IL-1β, and IL-18 mRNA **(A)** and protein **(B)** in lung of wild type mice, 24 h after single ozone exposure (1 ppm during 1 h). Representative data of two-independent experiments are shown. Results are expressed as mean ± SEM and are from one experiment with *n* = 4–6. Statistical test: Mann–Whitney, *p* value: *<0.05.

We first investigated whether ozone exposure augmented the expression of the IL-1 family, members, IL-1α, IL-1β, and IL-18, in WT mice by measuring mRNA and protein levels in lung. Pulmonary IL-1α and IL-18 mRNA were increased 24 h after ozone exposure, while IL-1β mRNA did not change (Figure 1A). Furthermore, IL-1α protein was increased in the lung 24 h after ozone exposure, but neither IL-1β nor IL-18 were augmented (Figure 1B).

Therefore, ozone exposure induced IL-1α mRNA and protein expression in the lung at 24 h, at a time when inflammation is clearly visible, but there was no significant difference for IL-1β or IL-18 protein expression.

### IL-1α Is Essential for Ozone Induced Lung Injury and Inflammation

**Figure 2:**
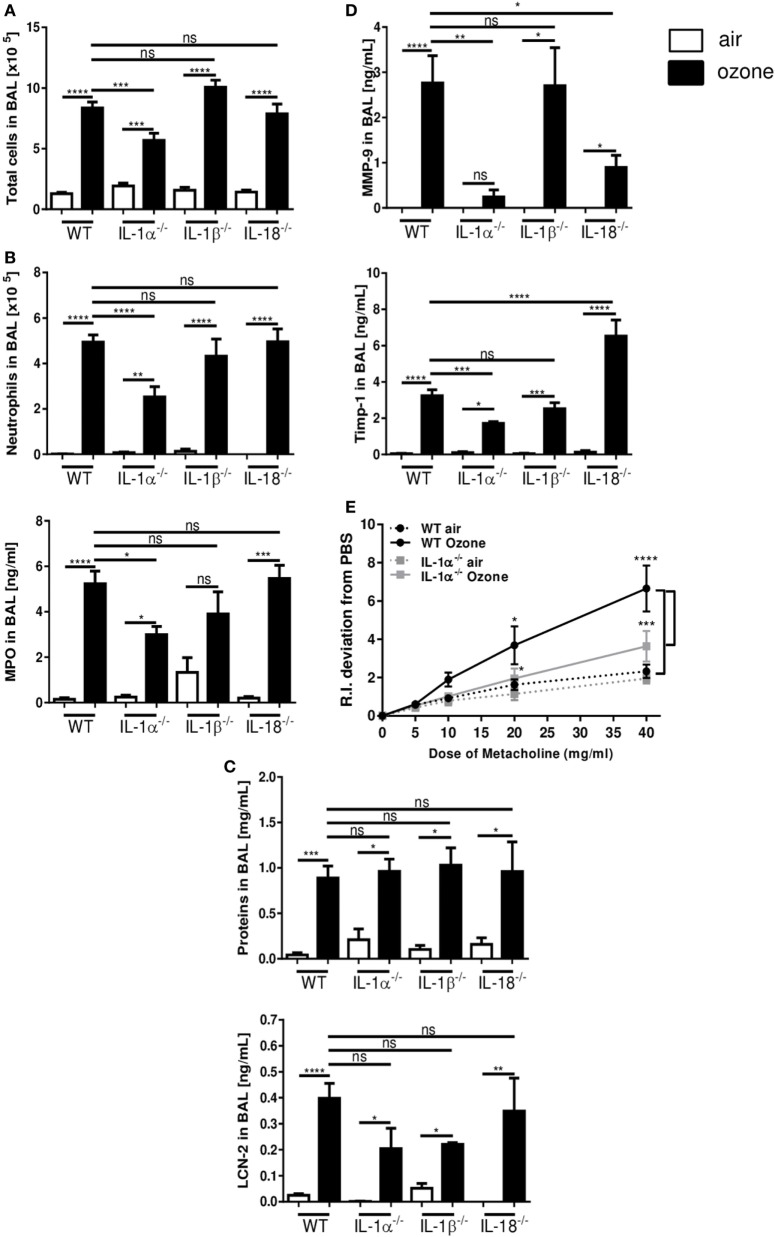

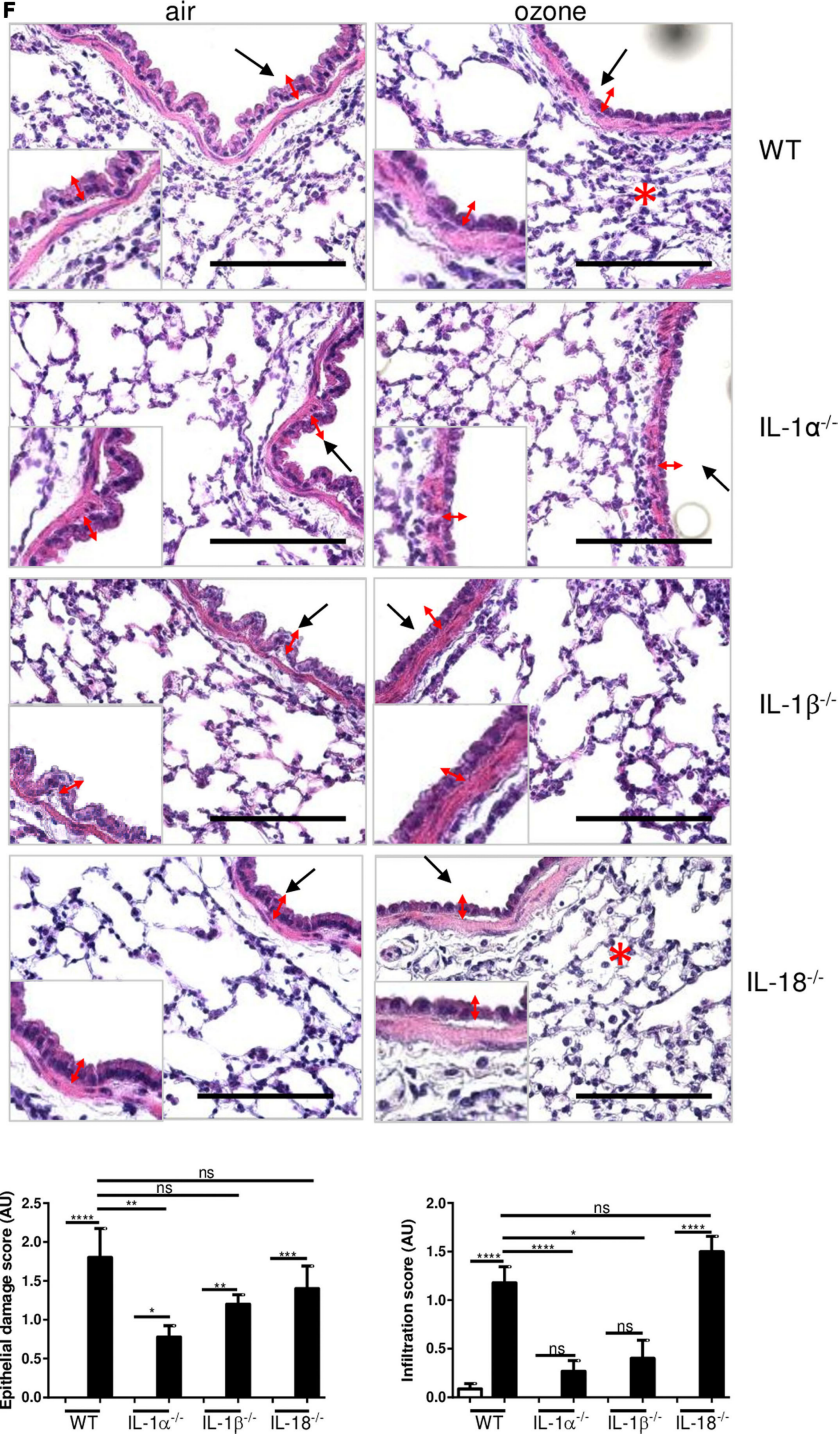
Inflammation in interleukin (IL-1)α^−/−^, IL-1β^−/−^, and IL-18^−/−^ mice 24 h after ozone exposure. Total cells **(A)**, neutrophils and myeloperoxidase **(B)**, protein and lipocalin-2 **(C)**, tissue remodeling parameters, tissue inhibitor metalloproteinase 1, and matrix metalloproteinase 9 **(D)**, histology, scale bar = 100 µm **(F)**, in IL-1α^−/−^, IL-1β^−/−^, and IL-18^−/−^ mice and airway hyperreactivity in IL-1α^−/−^ mice **(E)**, and microscopic analysis of the lung **(F)** at 24 h after ozone exposure. Representative data of two-independent experiments are shown. Results are expressed as mean ± SEM and are from one experiment, with *n* = 5–7. Statistical test: ordinary one-way ANOVA, with Bonferroni post test, *p* value: ****<0.0001, ***<0.001, **<0.01, *<0.05.

To address the respective role of the different IL-1 family members in ozone-induced lung inflammation we exposed IL-1α^−/−^, IL-1β^−/−^, and IL-18^−/−^ mice to ozone (1 ppm for 1 h). Total cell counts in BAL fluid were reduced in IL-1α^−/−^ mice, but not in IL-1β^−/−^ and IL-18^−/−^ mice in comparison to WT mice, 24 h after ozone exposure (Figure 2A). Further, ozone-induced recruitment of neutrophils and MPO levels in BAL fluid were diminished in the absence of IL-1α, but unaffected by the absence of IL-1β or IL-18 (Figure 2B). Protein extravasation in BAL fluid and lipocalin-2 (LCN-2) levels were not significantly affected by the absence of IL-1α, IL-1β, or IL-18 (Figure 2C). Further, ozone-induced tissue inhibitor metalloproteinase 1 (Timp-1) and matrix metalloproteinase 9 (MMP-9) expression were significantly reduced in IL-1α^−/−^, but not in IL-1β^−/−^ mice (Figure 2D). Conversely, in IL-18^−/−^ mice TIMP-1 was increased, while MMP-9 was reduced, as compared to ozone-exposed WT mice (Figure 2D). Ozone exposure causes airway hyperreactivity (AHR) in response to methacholine in WT mice (37). AHR was significantly reduced in IL-1α^−/−^ mice in comparison with WT after ozone exposure (Figure 2E). Histologically, the absence of IL-1α dampened the lung inflammatory cell infiltration and epithelial damage in the lung in response to ozone, but not in the absence of IL-18 (Figure 2F). As reported before (28), ozone caused acute cell death mainly of epithelial cells (Figures S2A–C in Supplementary Material). IL-1α deficiency reduced epithelial cell desquamation after ozone exposure (Figure S1B in Supplementary Material), while IL-1β^−/−^ mice presented diminished inflammation, but not for epithelial damage (Figure 2F). The data suggest that IL-1α-deficient mice are protected from ozone-induced epithelial injury and inflammation, but not IL-18 or IL-1β-deficient mice, suggesting that only IL-1α is involved in tissue injury and inflammatory response to ozone exposure.

### IL-1α Neutralization Reduced Inflammation Induced by Ozone Exposure

**Figure 3:**
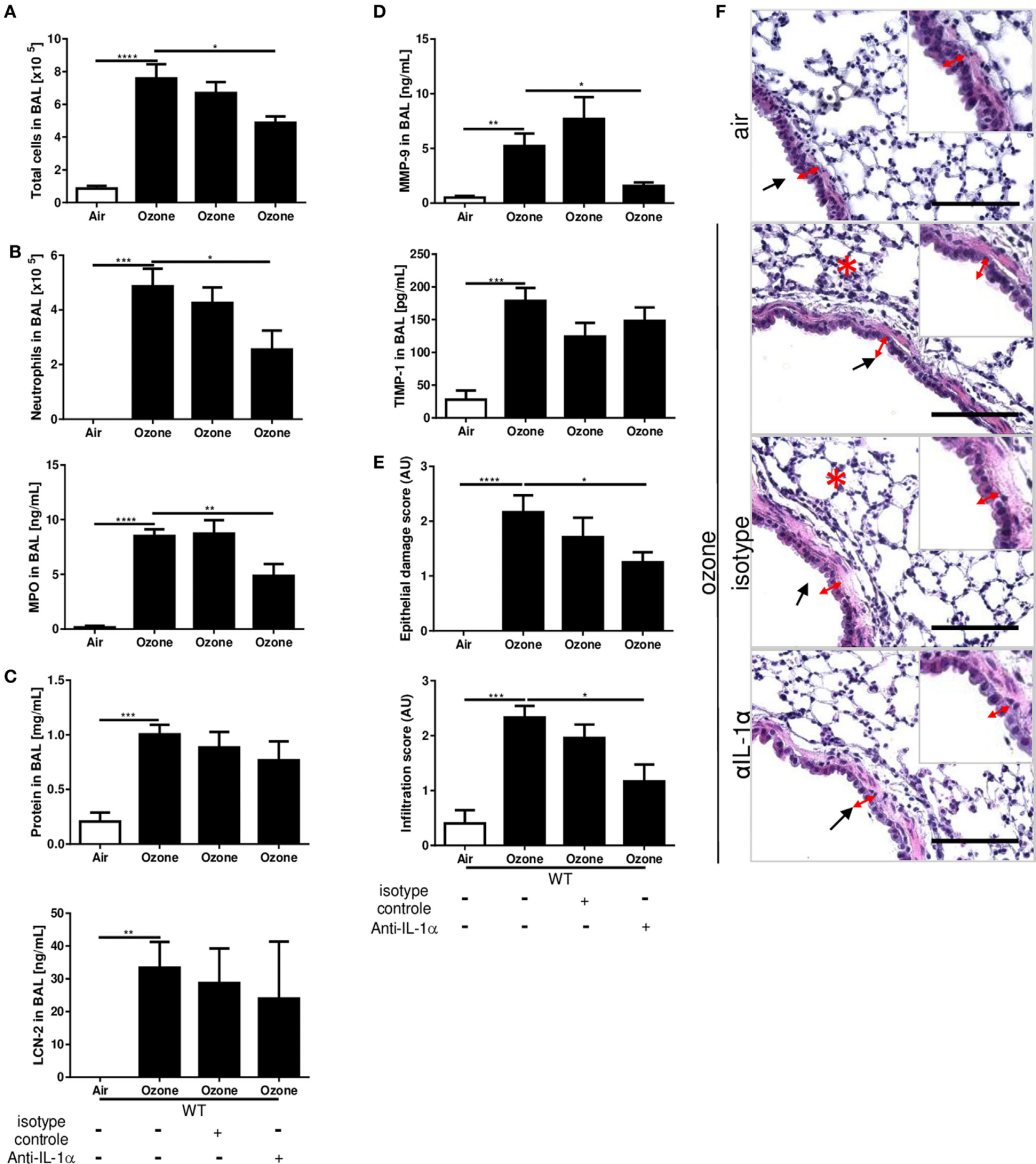
Interleukin-1 (IL-1)α antibody blockade reduces ozone-induced lung inflammation. A single administration of IL-1α neutralizing antibody 12 h before ozone exposure in wild type mice reduced inflammatory parameters. Total cell **(A)**, neutrophils (count and myeloperoxidase) **(B)**, total proteins and lipocalin-2 **(C)**, matrix metalloproteinase 9, and tissue inhibitor metalloproteinase 1, **(D)** and histology with epithelial damage and inflammatory score **(E)**, associated with histological picture **(F)**, scale bar = 100 µm, 24 h after ozone exposure. Representative data of two-independent experiments are shown. Results are expressed as mean ± SEM and are from one experiment, with *n* = 5–6. Statistical test: ordinary one-way ANOVA, with Bonferroni post test, *p* value: ****<0.0001, ***<0.001, **<0.01, *<0.05.

To confirm the role of IL-1α in airway inflammatory response we administered neutralizing IL-1α antibody (injection i.p. of 200 μg/mouse) 12 h before ozone exposure in WT mice. IL-1α antibody blockade (Figure S1C in Supplementary Material) diminished inflammation in BAL fluid with reduced total cell, neutrophil recruitment, and MPO levels in comparison to isotype control-treated mice (Figures 3A,B). This was associated with a small, but non-significant reduction of protein levels (Figure 3C). The remodeling protein MMP-9 was reduced after anti-IL-1α antibody treatment (Figure 3D), but not TIMP-1 (Figure 3D) and LCN-2 (Figure 3C) in BAL, while the administration of the isotype control antibody had no effect. Histologically, epithelial cell damage and inflammatory cell infiltration in the lungs were reduced by neutralizing IL-1α antibody (Figures 3E,F). Therefore, IL-1α antibody neutralization recapitulates the attenuated lung injury and inflammation response found in IL-1α-deficient mice as compared to WT control upon acute ozone exposure.

### Ozone-Induced Lung Inflammation Is MyD88-Dependent in Type 1 Alveolar Epithelial Cells

**Figure 4:**
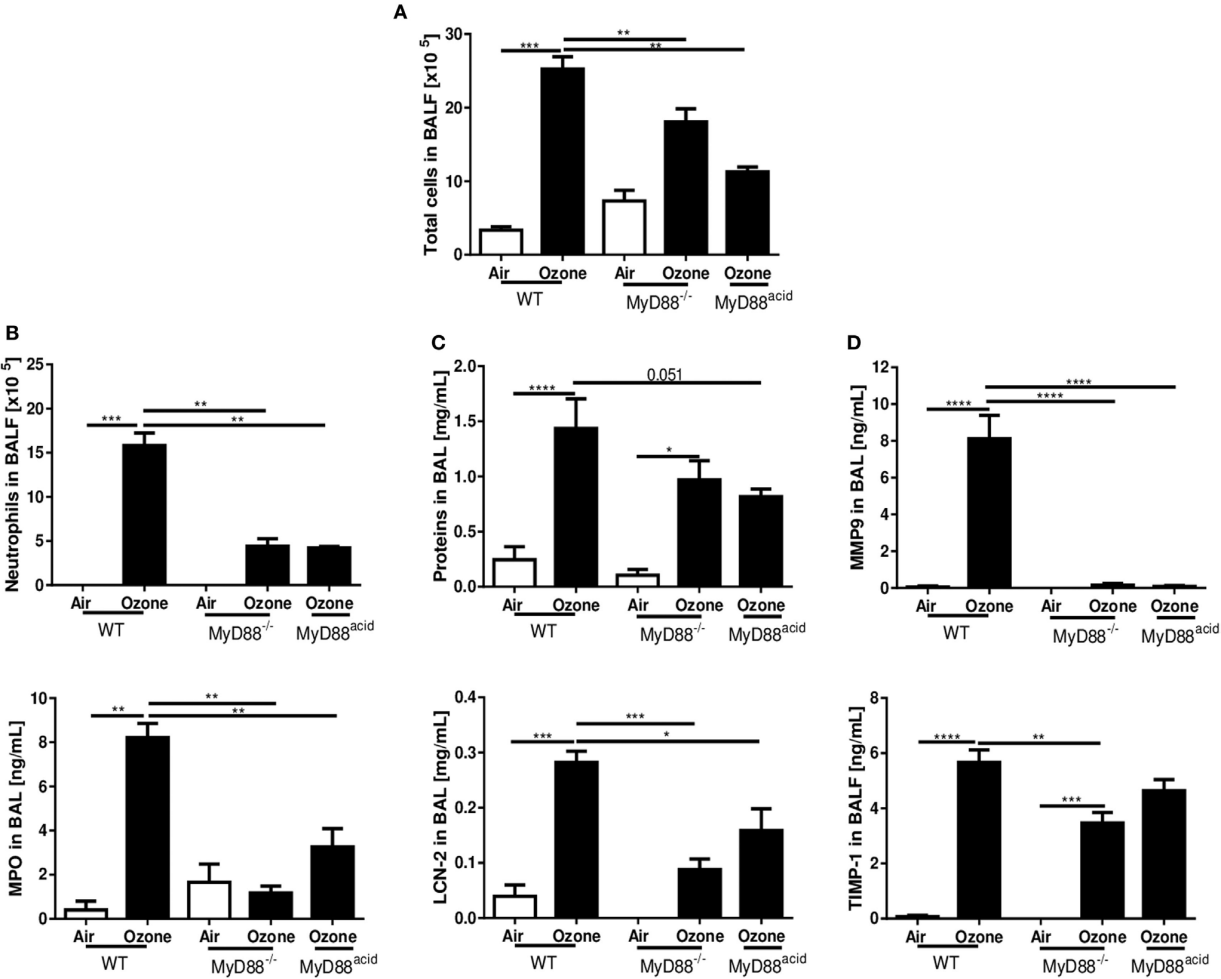

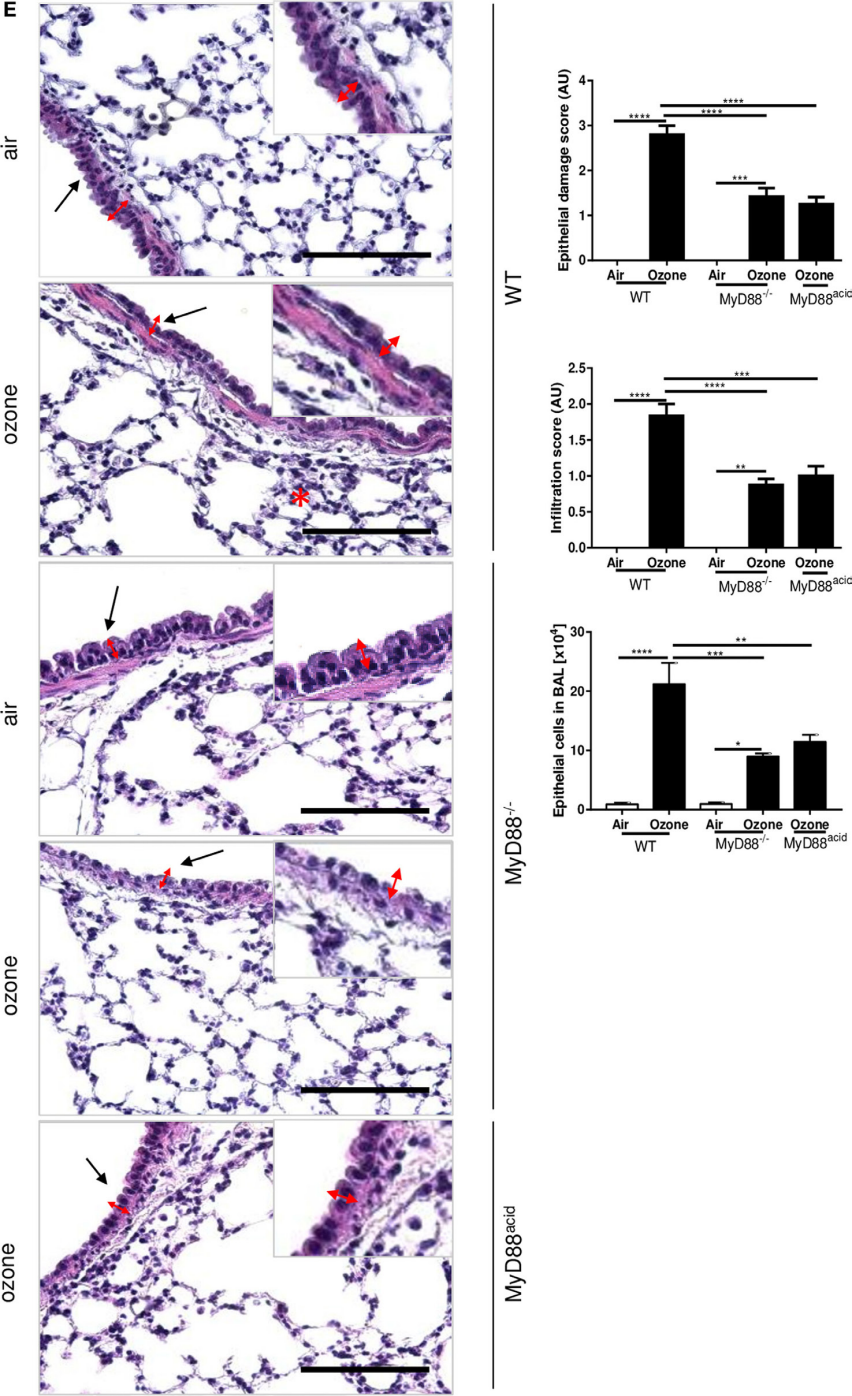
The myeloid differentiation factor-88 (MyD88) pathway in epithelial cell is involved in inflammation induced by ozone. Total cell number **(A)**, neutrophil counts and myeloperoxidase **(B)**, protein and lipocalin-2 **(C)**, tissue remodeling parameters, matrix metalloproteinase 9, and tissue inhibitor metalloproteinase 1 **(D)**, and histology, scale bar = 100 µm **(E)** are shown in wild type, MyD88^−/−^ and MyD88^acid^ mice, 24 h after ozone exposure. Representative data from two-independent experiments are given. Results are expressed as mean ± SEM and are from one experiment, with *n* = 5–6. Statistical test: ordinary one-way ANOVA, with Bonferroni post test, *p* value: ****<0.0001, ***<0.001, **<0.01, *<0.05.

We next asked whether MyD88 signaling is involved in the inflammation induced by acute ozone exposure. To answer this question, we exposed MyD88^−/−^ mice to ozone as before. Ozone-induced inflammation in MyD88^−/−^ mice which is attenuated with reduced total cells (Figure 4A), neutrophils, and MPO (Figure 4B) in BAL fluid, in comparison to WT mice at 24 h post-exposure. Moreover, total protein and LCN-2 (Figure 4C) and MMP-9 and TIMP-1 in BALF were reduced in MyD88^−/−^ mice in comparison with WT mice (Figure 4D), although the trend did not reach statistical difference for total protein in BAL. The histological investigations revealed that epithelial damage and inflammation were attenuated in MyD88^−/−^ mice (Figure 4E). Thus, absence of a functional MyD88 pathway largely prevented ozone-induced lung inflammation.

Since ozone disrupts the epithelial barrier with cell desquamation within 4 h after ozone exposure (28), we asked whether MyD88 signaling in epithelial cells is important for the injury response. We generated cell-specific MyD88-deficient mice for epithelial cells, MyD88^acid^ mice with inactivated MyD88 expression in type 1 lung epithelial cells (6). MyD88^acid^ mice exposed to ozone as above exhibited a reduced neutrophil recruitment, epithelial cell damage, protein leak, and tissue remodeling similar to complete MyD88^−/−^ mice (Figures 4A–E). Therefore, absence of MyD88 signaling in epithelial cells is sufficient to significantly reduce ozone-induced respiratory barrier injury and inflammation (Figure 4E).

**Figure 5:**
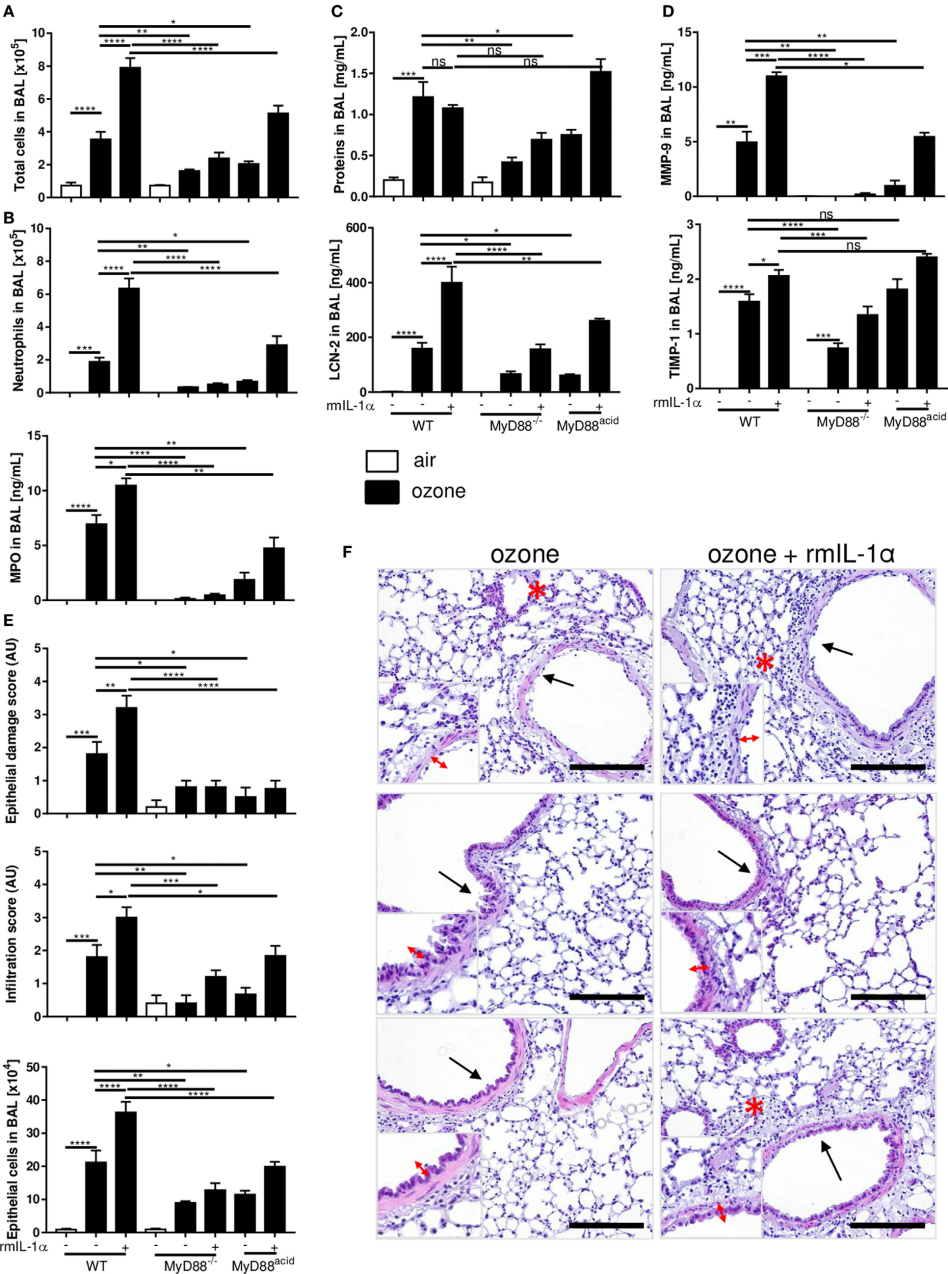
The myeloid differentiation factor-88 (MyD88) pathway in epithelial cell partially though interleukin-1α signaling is involved in inflammation and cell damage induced by ozone. Total cell number **(A)**, neutrophil counts and myeloperoxidase **(B)**, protein and lipocalin-2 **(C)**, tissue remodeling parameters, matrix metalloproteinase 9, and tissue inhibitor metalloproteinase 1 **(D)**, histological scoring, epithelial cells desquamation **(E)**, and histology, scale bar = 100 µm **(F)** are shown in wild type, MyD88^−/−^ and MyD88^acid^ mice, 24 h after ozone exposure. Results are expressed as mean ± SEM and are from one experiment, with *n* = 5–6. Statistical test: ordinary one-way ANOVA, with Bonferroni post test, *p* value: ****<0.0001, ***<0.001, **<0.01, *<0.05.

To test directly the IL-1α signaling in epithelial cells during ozone induced inflammation, we injected by i.t. mouse recombinant IL-1α (rmIL-1α) (0.8 µg by i.t.) in WT, MyD88^−/−^, and MyD88^acid^ mice. As expected rmIL-1α i.t. injection augmented the inflammatory response in WT mice, but less in MyD88^acid^ mice (Figures 5A–D). Furthermore, rmIL-1α elicited an increased of EPCAM^+^ epithelial cells recruitment in WT as compared to MyD88^acid^-deficient mice (Figure 5E).

Microscopic analysis of ozone-exposed lungs revealed that rmIL-1α increased epithelial cell damage and inflammatory cell infiltration in WT mice, which is attenuated in MyD88^acid^-deficient mice (Figures 5E,F).

Therefore, the data strongly suggest that IL-1α signaling in epithelial cells enhances ozone-induced lung injury and inflammation, which is largely reduced in the absence of MyD88 in lung epithelial cells.

## Discussion

We reported before that ozone causes respiratory epithelial damage, with desquamation and cell death (28) with the release of danger signals, also known as alarmins, such as IL-33, IL-1α, and others. Ozone-induced reactive oxygen species may activate the NLRP3 inflammasome leading to the maturation and release of IL-1 family members. The functional role of NLRP3 for inflammation after ozone exposure has been reported, but not confirmed (38).

Here, we report that ozone exposure is associated with increased IL-1α RNA and protein levels in the lung at 24 h after ozone exposure. IL-1α is produced by many cells types including epithelial cells and macrophages. We previously showed that IL-33, another alarmin, was present 4 h after ozone exposure, mostly expressed in epithelial cells and associated with cell death (28). We focused on epithelial cells, but other cells can be activated and/or undergo cell death, such endothelial, pericytes, smooth muscle cell, and recruited inflammatory cells. At 24 h, epithelial cells continued to produce IL-33, but other cell type release this cytokine, such as macrophages (28). Macrophages are also an established source of IL-1α and *in vitro* studies suggested that macrophage-derived IL-1α targets type I alveolar epithelial cells with the release of neutrophils attracting chemokines (19). By contrast, we did not find IL-1β message or protein expression at this time point.

However, increased IL-1β levels have been described previously after ozone challenge with a peak at 4 h (RNA) and 8 h (protein) (20, 21), but higher and longer ozone exposure (2 ppm for 3 h) were used. Park et al. reported a twofold increase of IL-1β mRNA upon exposure, and the administration of IL1ra (Anakinra), which competes with IL-1α and IL-1β, attenuated airway hyperreactivity and inflammation (21).

To address the functional relevance of IL-1α, IL-1β, and IL-18 in acute ozone-induced lung inflammation, we concomitantly used IL-1α^−/−^, IL-1β^−/−^, and IL-18^−/−^ mice. While deficiency of IL-1α attenuated protein leak and neutrophil recruitment into the BAL fluid of ozone-challenged mice, deficiency of IL-1β and IL-18 had no such effects. Likewise, only IL-1α^−/−^ mice had a significantly reduced expression of MPO, Timp-1, and MMP-9, airway hyperreactivity, and lung inflammation, while no effect was found in IL-1β^−/−^ mice. Therefore, IL-1α is critical in ozone-induced lung inflammation. Our results are consistent with the report on IL-1R1-dependent signaling of IL-1α using IL-1R1-deficient mice (22).

Furthermore, we questioned whether IL-18 is augmented, and found increased IL-18 mRNA, but no protein expression upon ozone exposure at 24 h. IL-18^−/−^ mice had no effect on protein leak, respiratory barrier damage, and lung inflammation had elevated Timp-1 levels and significantly aggravated lung inflammation score. These findings may suggest a potentially protective function of IL-18 for the lung, as discussed previously for epithelial barrier function in the intestine, whereas IL-18 was found to have both detrimental and beneficial functions (39).

Interleukin-1α binding to IL-1R1 signals *via* MyD88 (12). The MyD88 pathway is activated by different receptors, including IL-1R1 and IL-18R. Previous studies reported a role for TLR4 (10), MyD88 signaling (9), and IL-1 family members (20) in response to ozone. Here, we revisited the role of MyD88-dependent IL-1α signaling for lung inflammation in response to ozone exposure. We find that MyD88-deficient mice had a dramatic reduction of total cell and neutrophil recruitment into the BAL as well as a reduction in MPO, total protein, LCN-2, TIMP-1, and MMP-9 levels and diminished epithelial damage. Importantly, we show that MyD88 signaling in type I epithelial cells is critical for the inflammatory response, since genetic ablation of MyD88 in epithelial cells yields a similar reduction as observed in complete MyD88^−/−^ mice. These findings suggest a predominant role of MyD88 signaling in type I epithelial cells in promoting ozone-induced inflammation. We hypothesized that the alarmin IL-1α could be released by dying epithelial cells or macrophages and enhance epithelial cell death and ozone-induced lung inflammation. Our data using rmIL-1α demonstrate increased injury and inflammation, which is dependent on MyD88 expression in epithelial cells.

In conclusion, ozone causes epithelial barrier injury with the release of the alarmin IL-1α and leading to lung inflammation which is IL-1α-dependent signaling through MyD88. Importantly, epithelial cell signaling is crucial, since deletion of MyD88 in lung type I alveolar epithelial cells reduces ozone-induced inflammation. Therefore, epithelial cells may be critical target of the alarmin IL-1α upon acute ozone exposure leading to lung inflammation.

## Ethics Statement

Animal experiments were performed with the approval of the French Institutional Ethical Committee under agreement CLE CCO 2015-1088.

## Author Contributions

CM, DP, and BR designed experiments. CM, IM, and LF performed experiments. DP, CM, BR, CW, KC and VQ wrote the article.

## Conflict of Interest Statement

The authors declare that the research was conducted in the absence of any commercial or financial relationships that could be construed as a potential conflict of interest.

## Supplementary Material

Figure S1 Gating strategy, epithelial cells desquamation, and interleukin-1 (IL-1)α and IL-1Rα protein expression. Gatting strategy for epithelial desquamation analysis and cells mortality **(A)**. Epithelial cells desquamation after ozone exposure in wild type and IL-1α^−/−^ mice **(B)**. Analysis of IL-1α and IL-1Rα protein at 24 h after ozone exposure **(C)**. Representative data of two-independent experiments are shown. Results are expressed as mean ± SEM and are from one experiment, with *n* = 5–6. Statistical test: ordinary one-way ANOVA, with Bonferroni post test, *p* value: ****<0.0001, ***<0.001, **<0.01, *<0.05.

Figure S2 Cell mortality after ozone exposure. Gating strategy for cell death **(A)**. Cell death difference between air and ozone wild type (WT) mice **(B)**, epithelial cells mortality between WT, myeloid differentiation factor-88 (MyD88)^−/−^, MyD88^acid^, and interleukin-1α^−/−^ mice after ozone exposure **(C)**. Representative data of two-independent experiments are shown. Results are expressed as mean ± SEM and are from one experiment, with *n* = 5–6. Statistical test: ordinary one-way ANOVA, with Bonferroni post test, *p* value: ****<0.0001, ***<0.001, **<0.01, *<0.05.

## Abbreviations

AHR, airway hyperreactivity; BAL, bronchoalveolar lavage; KC, keratinocyte chemoattractant; ILC, innate lymphoid cells; LCN-2, lipocalin-2; MMP-9, matrix metalloproteinase 9; MPO, myeloperoxidase; ROS, reactive oxygen species; TIMP-1, tissue inhibitor metalloproteinase 1; WT, wild type/C57BL6 mice.
