# Supplementary figures and images for: Interleukin-1α Mediates Ozone-Induced Myeloid Differentiation Factor-88-Dependent Epithelial Tissue Injury and Inflammation
Source: Front Immunol. 2018 May 7;9:916. doi: 10.3389/fimmu.2018.00916 (PMC5950844; doi:10.3389/fimmu.2018.00916)

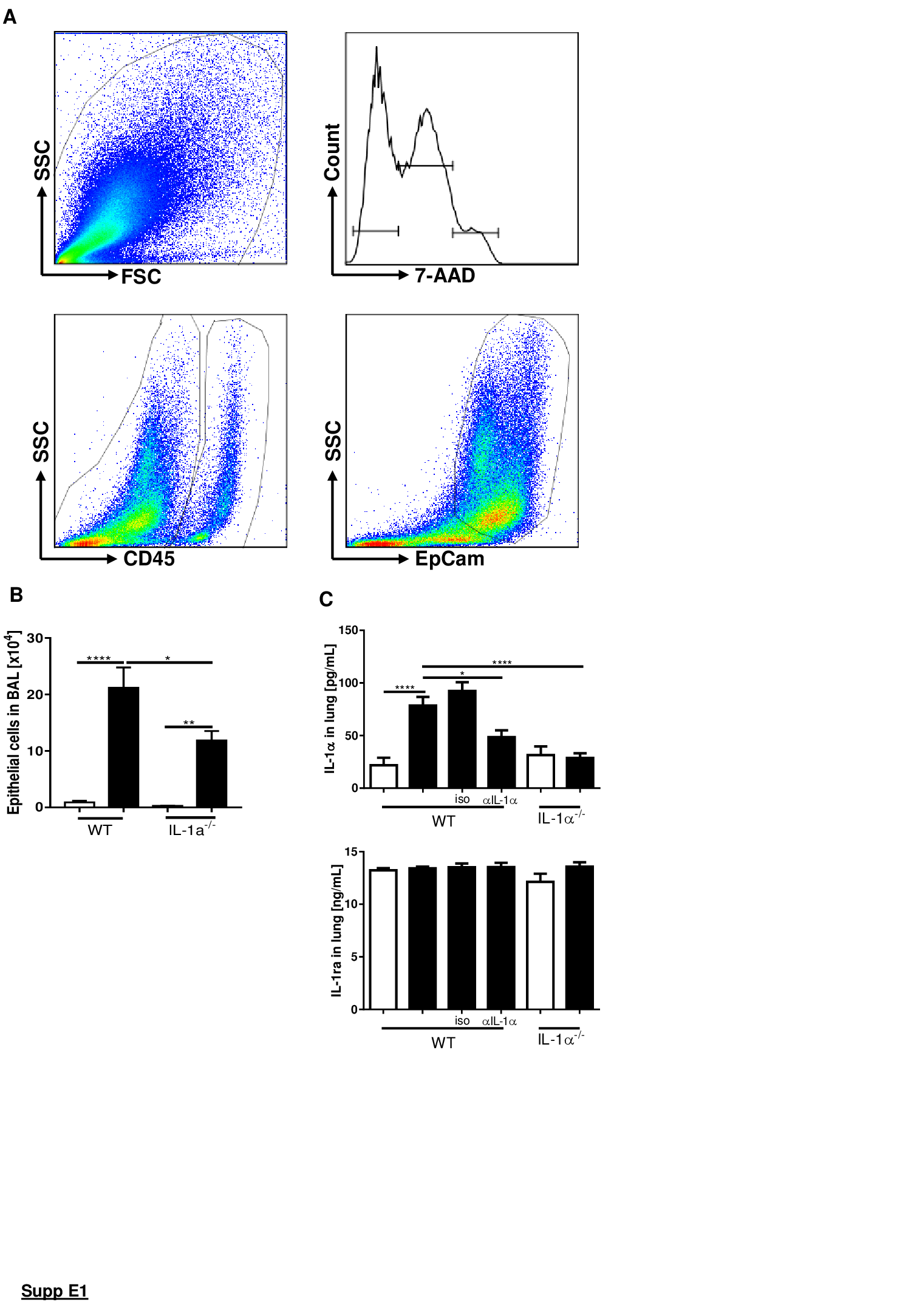

Supplement: Figure S1 — Gating strategy, epithelial cells desquamation, and interleukin-1 (IL-1)α and IL-1Rα protein expression. Gatting strategy for epithelial desquamation analysis and cells mortality (A). Epithelial cells desquamation after ozone exposure in wild type and IL-1α−/− mice (B). Analysis of IL-1α and IL-1Rα protein at 24 h after ozone exposure (C). Representative data of two-independent experiments are shown. Results are expressed as mean ± SEM and are from one experiment, with n = 5–6. Statistical test: ordinary one-way ANOVA, with Bonferroni post test, p value: ****<0.0001, ***<0.001, **<0.01, *<0.05. [file image_1.tiff]

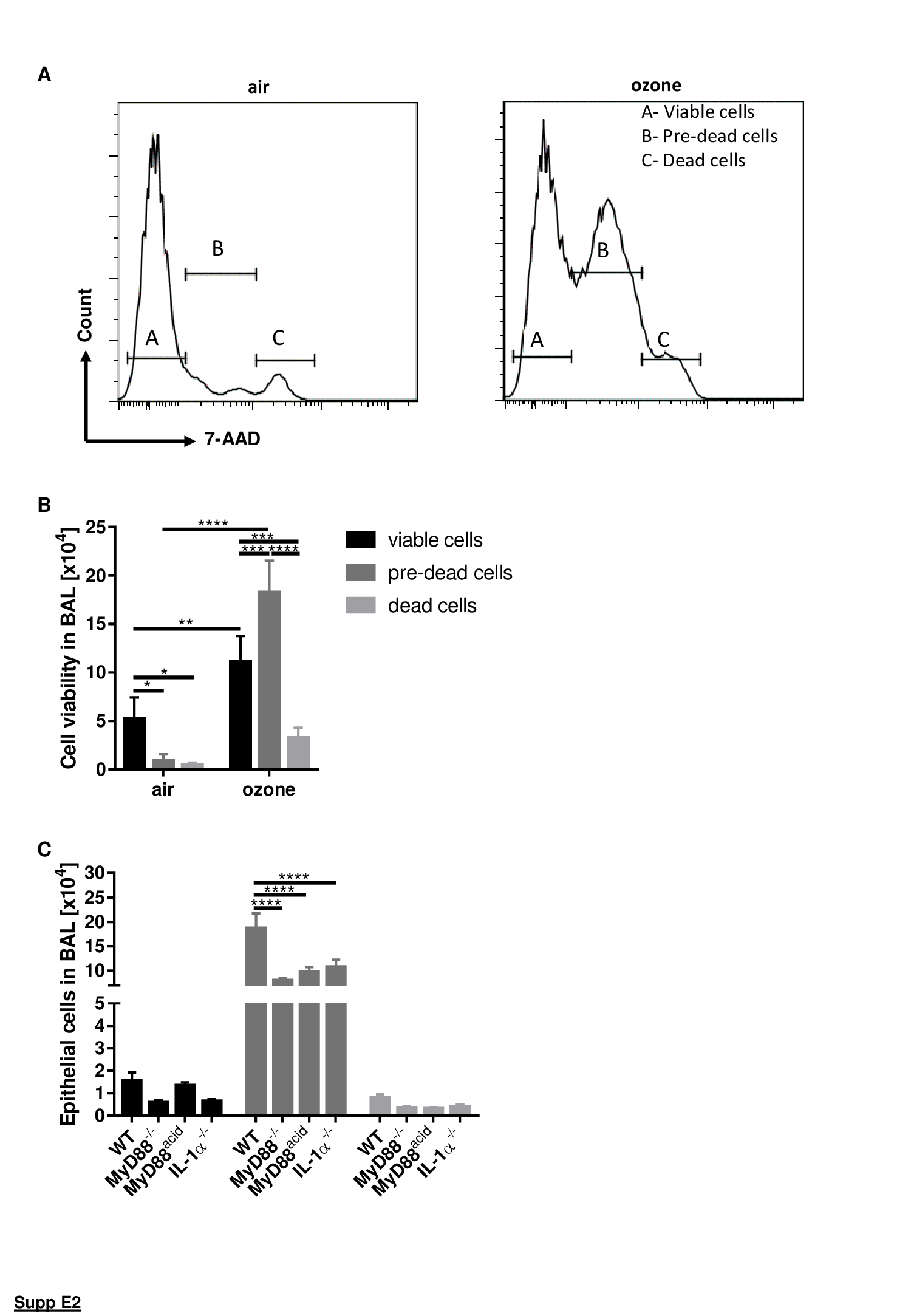

Supplement: Figure S2 — Cell mortality after ozone exposure. Gating strategy for cell death (A). Cell death difference between air and ozone wild type (WT) mice (B), epithelial cells mortality between WT, myeloid differentiation factor-88 (MyD88)−/−, MyD88acid, and interleukin-1α−/− mice after ozone exposure (C). Representative data of two-independent experiments are shown. Results are expressed as mean ± SEM and are from one experiment, with n = 5–6. Statistical test: ordinary one-way ANOVA, with Bonferroni post test, p value: ****<0.0001, ***<0.001, **<0.01, *<0.05. [file image_2.tiff]
